# Calciphylaxis: A Long Road to Cure with a Multidisciplinary and Multimodal Approach

**DOI:** 10.1155/2022/3818980

**Published:** 2022-06-08

**Authors:** Vasiliki Zoi, Dimitra Bacharaki, Aggeliki Sardeli, Minas Karagiannis, Sophia Lionaki

**Affiliations:** Department of Nephrology, National and Kapodistrian University of Athens, Attikon Hospital, Athens, Greece

## Abstract

Calciphylaxis is a rare yet potentially fatal condition, resulting from ectopic calcification of the small arterioles of the dermis with resulting necrotic lesions infection, sepsis, and death. In hemodialysis patients, its prevalence ranges between 1 and 4%, while mortality amounts to 30–80%. We present in here a 45-year-old female on chronic dialysis with morbid obesity, who was admitted for painful nodules in the lower abdomen and necrotic lesions at the lower extremities. Severe uremia and uncontrolled secondary hyperparathyroidism were the main characteristics in this patient, and thus, a clinical diagnosis of calciphylaxis was made. Treatment modalities included wound care plus antibiotics and analgesics, daily hemodialysis, and strategies targeting calcification with sodium thiosulfate, cinacalcet, and non-calcium-containing binders. A crucial factor for overcoming the infection-lesion vicious circle is thorough and daily care of the lesions. Nursing attention was focused on the motivation of her self-care, for the prevention of institutionalization and the psychological support of the patient and her family. The most intriguing feature was the fact that she experienced several exacerbations during the follow-up time. During the final relapse, she was prescribed hyperbaric oxygen sessions that actually put the disease under control thereafter. The good outcome for this patient was probably related to the combination of close follow-up along with a multidisciplinary approach.

## 1. Introduction

Calciphylaxis or uremic arteriolopathy (CUA) is a rare necrotic lesion of dermal and subcutaneous tissue resulting from ectopic calcification of the medial layer of arterioles and small arteries. These changes cause tissue ischemia, necrosis, ulceration, and finally sepsis. It was first described in 1968 by Anderson et al. as a necrotic lesion of dermal and subcutaneous tissue in patients with hyperparathyroidism and hypercalcemia [1]. Calciphylaxis primarily affects patients on dialysis or after renal transplantation; however, exceptions have been reported. The average age of onset is between 50 and 60 years with a clear female predominance [2, 3]. Major risk factors include Caucasian ethnicity, female gender, diabetes mellitus, dialysis, obesity, hypoalbuminemia in the context of malnutrition and chronic inflammation, hyperphosphatemia, elevated calcium x phosphate product, and combined use of active vitamin D analogues with high doses of calcium-containing phosphate binders [4, 5] and the use of vitamin K antagonists. Among hemodialysis patients, the incidence rate has been estimated at 35 cases per 10,000 patients in the United States and 4 cases per 10,000 patients in Europe. The mortality rate however amounts to 30–80% and is more often related to infectious complications resulting in sepsis. Furthermore, the overall one-year survival does not exceed 40% [6]. Clinically, it frequently develops in the lower extremities but may also manifest around the abdomen and hips and may affect peripheral sites or even skeletal muscle. Management of CUA is primarily based on wound care with surgical debridement and systemic antibiotics, elimination of all possible precipitating factors of ectopic calcification, administering agents capable of inhibiting the progress of calcification, and controlling risk factors to avoid recurrences.

We describe in here a patient with calciphylaxis, who experienced multiple relapses for more than a year necessitating the application of all available treatments. In our experience, patient survival was probably related to close monitoring, personalized medicine, and exploitation of a multidisciplinary strategy [1, 7].

## 2. Case Description

**Figure 1:**
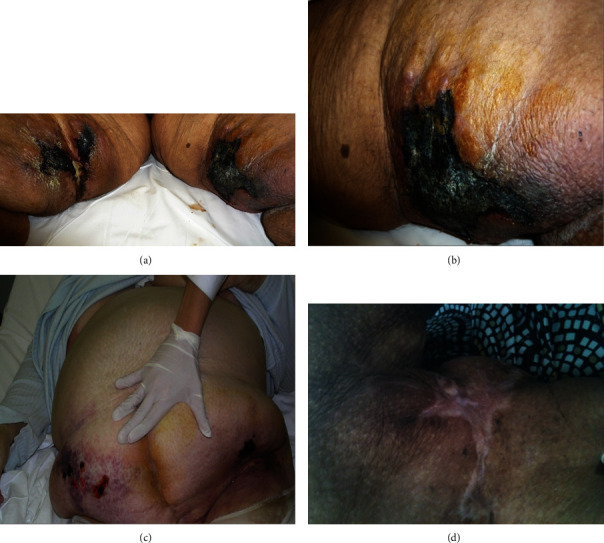
(a) Necrotic tissues of the thighs; (b) necrotic tissues; (c) abdomen ulcers; (d) healed necrotic lesions with scars.

A 45-year-old obese female on hemodialysis presented to the emergency department with extremely painful nodules of the lower abdomen area and necrotic lesions at the buttocks and inner surface of the thighs (Figures 1(a)–1(c)). She had been dialyzed for two months, through a temporary central venous catheter, that was malfunctioning. She had poorly controlled Hashimoto thyroiditis and suffered from morbid obesity (BMI: 53 Kg/m^2^). Laboratory results at presentation were suggestive of uncontrolled uremia and severe hyperparathyroidism (Table 1). Notably, the product of phosphate x calcium (Ca) was 81.34 with high serum parathormone (PTH) of 1500 pg/*μ*L, but ultrasonography of the parathyroid area was negative for adenomas.

**Figure 2:**
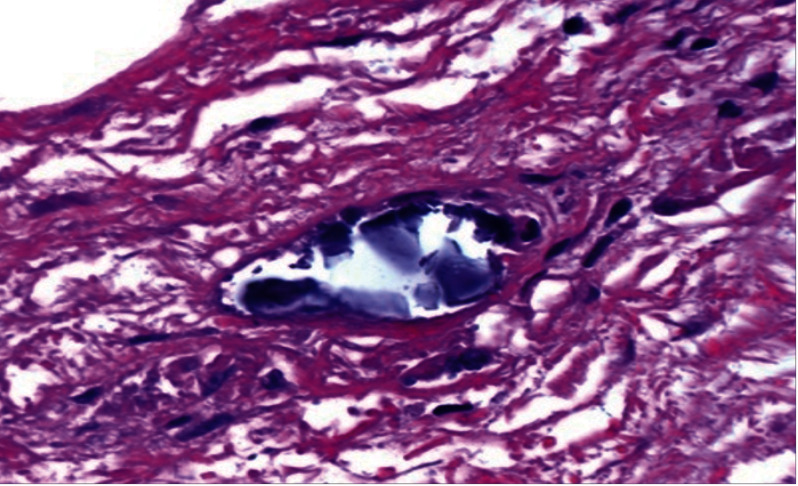
Skin biopsy.

A definitive diagnosis of calciphylaxis was made by a skin biopsy (Figure 2), which revealed medial calcification of dermal arterioles or small arteries and fibrointimal hyperplasia, microthrombi, and vascular narrowing or occlusion, often with evidence of necrosis. Calcification of small- to medium-sized blood vessels was also seen. The intimal layer of blood vessels was significant for fibrosis, and intravascular thrombi were seen. The main feature was the diffuse calcification of small capillaries in the adipose tissue.

**Figure 3:**
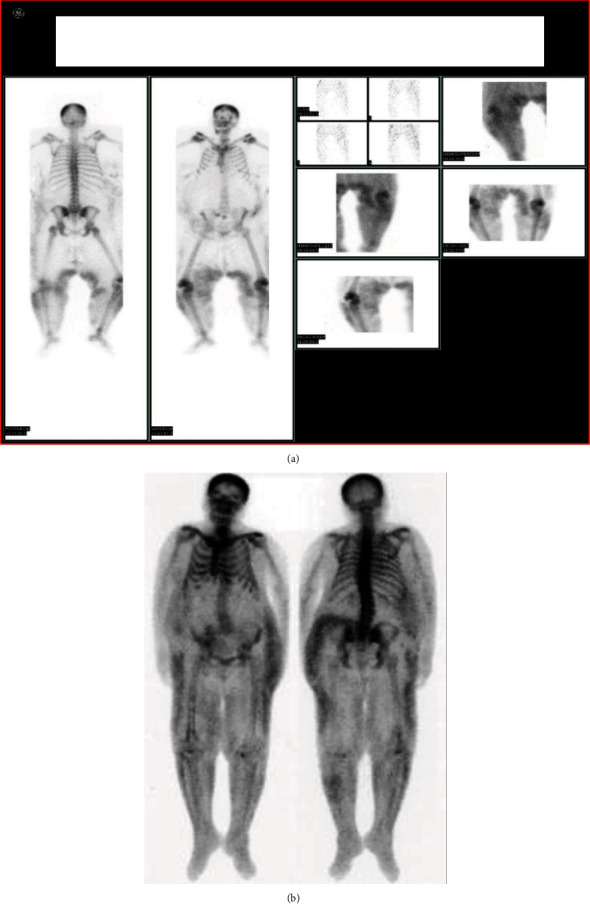
(a) Bone scintigraphy with technetium 99 m before therapy; (b) bone scintigraphy with technetium 99 m after therapy.

A nuclear bone scan (Figure 3(a)) using scintigraphy with technetium 99 m revealed increased radiotracer uptake in the soft tissue of the thighs, homogenously and bilaterally, as well as in all areas of the skin lesions. Notably, a three-phase technetium 99 m methylene diphosphate bone scan is not required for the diagnosis of CUA but it may be a helpful adjunct, especially in early lesions, since it is highly sensitive and specific to identify the extent of cutaneous calcifications. In this case, bone scintigraphy was helpful in order to demonstrate the response to treatment (as decreased radiotracer uptake).

### 2.1. Management and Outcome

Treatment strategies aimed to maintain phosphorus levels at less than 5.5 mg/dL. Dialysis frequency was increased (six times per week for 3-4 hours daily) to optimize the clearance of uremic molecules and improve bone-mineral markers while the central venous catheter was replaced by a permanent one. Calcium supplements and calcium-based phosphate binders were discontinued and converted to non-calcium-containing binders (800 mg, 3 TID). High calcium dialysate baths (greater than 2.5 mEq/L) were avoided. In order to manage hyperparathyroidism, cinacalcet, a calcimimetic agent, was introduced. Sodium thiosulfate was given intravenously at the end of hemodialysis, in low doses at first to inhibit soft tissue calcification. Side effects of sodium thiosulfate included metabolic acidosis and gastrointestinal disturbances such as nausea and vomiting. However, the patient was thoroughly informed and supported, in order to accept the treatment in carefully incremented doses.

Skin lesions were challenging to treat, with wound care and pain management being extremely important. Debridement was utilized to remove devitalized and necrotic tissue, prevent infection, and promote healing. Appropriate antibiotics were given culture-guided, regularly, and by clinical indication. During the final relapse, she was offered hyperbaric oxygen therapy to facilitate wound healing. Specifically, she underwent 10 sessions of hyperbaric oxygen sequentially and then once monthly, leading to completely healed lesions. The patient was reluctant to follow the recommendation for a minimum of 20 treatments, i.e., 5–7 sessions per week. However, we believe that this treatment played a tremendous role in the final outcome. Eventually, all the ulcers were replaced by a new epithelium (Table 2).

Nursing care included precise administration of the medication and handling of the increased incidents during hemodialysis, including vomiting due to the thiosulfate treatment. The new permanent central venous catheter was cared for on a daily basis. The treatment included the patient's immense pain to be cured with narcotic analgesic medication, and the lesions required daily cleaning. To avoid institutionalization, efforts were made to promote her self-care, motivate her, and prevent hospitalization's depression. She was assisted to keep in touch with her work and friends through telephone and internet and restart one of her hobbies, which was knitting. Gradually, she was able to bathe herself, get off the bed on her one, and walk through the hospital. Great deal of time was given for psychological support of the patient and her family with the assistance of a psychologist, as this ensured their compliance to the treatment and their full support. Additional care was paid by the nursing staff and the dietician to not skip meals, since adequate nutrition is imperative for wound healing. Pain of the lesions which was accentuated during hemodialysis, a characteristic feature of calciphylaxis, already made her anorectic. She was finally discharged 70 days after admission, but she was closely followed up for a total of 13 months. At that point, she had also lost 30 kg and had regained ambulatory capacity with regular physical therapy. Nowadays, she receives hemodialysis treatment with no relapses in terms of calciphylaxis (Figures 1(d) and 3(b)).

## 3. Discussion

Calciphylaxis is a rare, potentially life-threatening syndrome characterized by progressive and painful skin ulcerations associated with medial calcification of medium-sized and small cutaneous arterial vessels. Superinfection of necrotic skin lesions with subsequent sepsis significantly contributes to this outcome and, most of the time, leads to death [4]. This case of calciphylaxis was dominated by multiple relapses for more than a year, which were successfully managed using a personalized approach with multiple therapeutical modalities including increased dialysis frequency, non-calcium-containing binders, cinacalcet, sodium thiosulfate, and hyperbaric oxygen therapy. We considered organizing a clinical trial, but we did not pull through, due to low enrollment.

In most occasions, calciphylaxis is a clinical diagnosis. The biological content includes secondary hyperparathyroidism and obvious phosphorus and calcium metabolism disorders. Although pathogenesis remains incompletely understood, elevated calcium x phosphate product, increased serum PTH, warfarin, iron therapy, corticosteroids, and administration of activated vitamin D have been associated with the development of calciphylaxis. Yet, abnormalities of bone-mineral parameters are not sufficient to cause calciphylaxis on their own in most patients [9, 15]. Deficiency of vascular calcification inhibitors such as fetuin-A, osteoprotegerin, and matrix G1a protein may play a role in the development of calciphylaxis. Fetuin-A is a glycoprotein that binds calcium and phosphorus, and it is downregulated in dialysis patients. Osteoprotegerin plays an important role in bone metabolism. Matrix G1a protein may also prevent vascular calcification and is dependent on vitamin K-dependent carboxylation for its activity [10, 16].

Thiosulfate is a chelating agent, with high affinity to calcium ions, which may interfere with calcium and phosphate precipitation producing soluble calcium thiosulfate which can potentially be removed by dialysis [12, 13]. It may also interfere with the local inflammation process by antioxidant properties [17]. Treatment with sodium thiosulfate is off-label but has been routinely used in calciphylaxis and is considered the cornerstone of its treatment [12, 13]. The mechanism by which sodium thiosulfate treats calcific uremic arteriolopathy remains unknown. Previously, it was believed to work only through calcium chelation; however, recent studies suggest that sodium thiosulfate may also inhibit soft tissue calcification through mechanisms independent of calcium binding.

Moreover, Basile et al. [18] reported successful hyperbaric oxygen therapy in a small number of calciphylaxis patients. This approach is based on the attempt to improve wound healing in ischemic tissues. In that study, the patients were exposed to 100% oxygen under 2.5-fold elevated atmospheric pressure in a closed chamber for 90 minutes per session in order to significantly increase local oxygen pressure in the ulcerated and necrotic areas (the number of sessions per patient ranged between 20 and 108) while 8 of 11 patients showed effective healing of ulcerations [8, 17]. Finally, management of calciphylaxis aims at the restoration of phosphocalcic balance, effective detoxification of affected areas, and the elimination of as many risk factors as possible. Cinacalcet was introduced to lower PTH in place of activated vitamin D in order to control serum calcium and phosphorus levels [11].

In conclusion, the majority of cases with calciphylaxis is seen in the setting of end-stage kidney disease, as a result of secondary hyperparathyroidism and elevation of the serum calcium-phosphate product. A multifactorial etiology is likely. In this case, factors such as morbid obesity, inadequate dialysis, uncontrolled hyperparathyroidism, and history of vitamin D deficiency were possibly involved. Various treatment options were employed. Supportive management methods like pain control and wound care with sodium thiosulfate, hyperbaric oxygen therapy, tissue plasminogen activator, vitamin D and vitamin K supplementation, parathyroidectomy, and daily hemodialysis have been described. Sodium thiosulfate is thought to work by enhancing the solubility of calcium phosphate and removing calcifications. Importantly, a multidisciplinary approach is often needed to fight calciphylaxis including input from a nephrologist, dermatologist, dietician, wound surgeon, wound nurse, dialysis nurse, pain management specialist, palliative care team, and hyperbaric oxygen provider combined with close follow-up and escalation of treatment choices in autoimmune diseases.

## Figures and Tables

**Table 1:** Laboratory measurements at different points during the follow-up time.

Laboratory outcomes (serum)	On admission	1^st^ month	6^th^ month	End of follow-up
WBC (K/*μ*l)	14.41 K/*μ*l	7.33	12.81	8.05
NEUT. (K/*μ*l)	11.71	4.76	8.96	4.52
Hct (%)	22.3	28.7	29.9	27.5
Hb (g/dl)	6.6	8.6	8.7	9.1
Ca (mg/dl)	8.3	9.3	8.9	8.5
Urea (mg/dl)	227	92	34	32
Creatinine (mg/dl)	11.3	3.5	3	3.4
P (mg/dl)	9.8	3.6	2.4	2.7
PTH (pg/ml)	1500	48.6	45	42
CRP (mg/dl)	288	34.7	59.6	15

**Table 2:** Therapies used for the management of patients with calciphylaxis and mechanisms of action.

Therapies	Mechanism of action
Increased dialysis frequency	Clearance of uremic molecules [3, 4, 8]
Dialyzate calcium 1,25 mmol/L	Slowing down calcification [3, 4, 6]
Non-calcium-containing binders	Avoiding hypercalcemia—high phosphorus [3, 4, 9]
Cinacalcet	Controlling parathyroid hormones [10, 11]
Sodium thiosulfate	Inhibiting calcification [12, 13]
Debridement	Ulceration treatment [6]
Hyperbaric oxygen therapy	Tissue oxygenation—wound healing [8, 14]

## Data Availability

All data are included within the article.
